# The Potential Adjuvanticity of CAvant^®^SOE for Foot-and-Mouth Disease Vaccine

**DOI:** 10.3390/vaccines9101091

**Published:** 2021-09-28

**Authors:** Young-Hoon Ahn, W. A. Gayan Chathuranga, Young-Jung Shim, D. K. Haluwana, Eun-Hee Kim, In-Joong Yoon, Yong-Taik Lim, Sung Ho Shin, Hyundong Jo, Seong Yun Hwang, Hyun Mi Kim, Min Ja Lee, Jong-Hyeon Park, Sung-Sik Yoo, Jong-Soo Lee

**Affiliations:** 1 Choong Ang Vaccine Laboratory Co., Ltd., Daejeon 34055, Korea; hi-ayh@cavac.co.kr (Y.-H.A.); younggirl58@cavac.co.kr (Y.-J.S.); ehkim@cavac.co.kr (E.-H.K.); iyoon@cavac.co.kr (I.-J.Y.); 2 College of Veterinary Medicine, Chungnam National University, Daejeon 34314, Korea; gayachathu123@gmail.com (W.A.G.C.); dhammikaaz@gmail.com (D.K.H.); 3 Department of Nano Engineering, Sungkyunkwan University, Suwon 16419, Korea; yongtaik@skku.edu; 4 Animal and Plant Quarantine Agency, Gimcheon 39660, Korea; ikarus121@korea.kr (S.H.S.); jhd0327@korea.kr (H.J.); hsy8592@korea.kr (S.Y.H.); khm852456@korea.kr (H.M.K.); herb12@korea.kr (M.J.L.); parkjhvet@korea.kr (J.-H.P.)

**Keywords:** foot-and-mouth disease virus, vaccine, adjuvant, water-in-oil emulsion, CAvant^®^SOE

## Abstract

Foot-and-mouth disease (FMD) is a notifiable contagious disease of cloven-hoofed mammals. A high potency vaccine that stimulates the host immune response is the foremost strategy used to prevent disease persistence in endemic regions. FMD vaccines comprise inactivated virus antigens whose immunogenicity is potentiated by immunogenic adjuvants. Oil-based adjuvants have clear advantages over traditional adjuvant vaccines; however, there is potential to develop novel adjuvants to increase the potency of FMD vaccines. Thus, we aimed to evaluate the efficacy of a novel water-in-oil emulsion, called CAvant^®^SOE, as a novel vaccine adjuvant for use with inactivated FMD vaccines. In this study, we found that inactivated A22 Iraq virus plus CAvant^®^SOE (iA22 Iraq-CAvant^®^SOE) induced effective antigen-specific humoral (IgG, IgG1, and IgG2a) and cell-mediated immune responses (IFN-γ and IL-4) in mice. Immunization of pigs with a single dose of iA22 Iraq-CAvant^®^SOE also elicited effective protection, with no detectable clinical symptoms against challenge with heterologous A/SKR/GP/2018 FMDV. Levels of protection are strongly in line with vaccine-induced neutralizing antibody titers. Collectively, these results indicate that CAvant^®^SOE-adjuvanted vaccine is a promising candidate for control of FMD in pigs.

## 1. Introduction

Foot-and-mouth disease (FMD) is a highly contagious, economically important transboundary animal disease of cloven-hoofed animals such as cattle, swine, goats, and sheep [1]. The etiological agent, FMD virus (FMDV), is a positive-sense single-stranded RNA virus belonging to the genus Aphthovirus within the family Picornaviridae [2]. FMD causes severe economic losses due to loss of productivity, costly eradication policies, and impediments to international trade [3,4]. It was the first disease to be given official status recognition by the International Organization of Animal Health (OIE) [5].

Vaccination is a key strategy used to combat and eradicate FMD, particularly in enzootic areas [6]. Despite rapid and ongoing progress in the field of FMD vaccinology, most usable vaccines comprise mainly tissue culture-propagated, binary ethyleneimine (BEI)-treated, inactivated virus antigens, which are purified by ultrafiltration, polyethylene glycol precipitation, or chromatography [7,8,9]. In general, prophylactic inactivated vaccines used in endemic areas are concentrated to yield the equivalent of three 50% protective doses (PD_50_). Additionally, inactivated antigens concentrated to six PD_50_ or above have been used as an emergency vaccine to control outbreaks in FMD-free countries; this formulation was effective as early as 4 days post-vaccination (dpv) [10,11,12]. The Food and Agriculture Organization and the OIE both recommended strict guidelines for quality control testing of inactivated FMD antigen production; these guidelines pertain to identification, sterilization, safety profiles, potency, efficacy, and detection of FMDV non-structural proteins [13]. Production of inactivated FMD vaccines is strictly limited to biosafety level III containment facilities [10], which makes a high antigen payload almost impossible. Additionally, inactivated virus antigen alone is poorly immunogenic; thus inactivated FMD vaccines are regularly formulated with an effective adjuvant to increase immunogenicity while at the same time reducing the antigen payload [14,15,16].

The first field-level FMDV vaccine adjuvant (aluminum salt) was reported in 1937 [17]. Since then, diverse classes of compounds have been assessed as adjuvants. These include mineral salts, emulsions, saponins, microbial products, cytokines, polymers, and small molecules [18]. Though many types of adjuvant have been investigated, only two types (oil-based adjuvants or aqueous aluminum hydroxide [Al(OH)3] plus saponin) are used widely for field-level applications [19,20]. However, the oil-based adjuvant vaccines have several distinct advantages over traditional alum adjuvant vaccines. The early induce stronger and longer-lasting antibody responses, resulting in more effective protection [21,22,23]. Oil adjuvant vaccines are more effective in swine, which are referred to as the “amplifier hosts” of FMD [24,25]. Unlike alum adjuvant vaccines, oil adjuvant vaccine-derived immunity shows less interference from colostrum antibodies [26,27,28]. Additionally, the oil adjuvant enables the slower release of antigen via depot formation at the site of injection, and it is a more effective vehicle for transporting antigen throughout the lymphatic system while at the same time stimulating antigen-presenting cells [18]. In particular, recent candidates belonging to the Montanide^TM^ ISA series oil-adjuvants show superior efficacy when used with inactivated FMD vaccines; this formulation induces earlier and higher neutralizing antibody titers, stronger cellular immune responses, and greater protective efficacy in different susceptible animal species [14,29,30]. Although currently available inactivated whole antigen vaccines formulated with oil emulsion have been shown better efficacy to control the disease, continuous FMD outbreaks in countries like South Korea even under routing vaccination practices demonstrated the clear need of further improvement of current FMDV vaccine platform, this has created a major need for improved and more powerful adjuvants for use in the FMD vaccines.

In this study, we evaluated the efficacy of novel water-in-oil (W/O) emulsion adjuvant CAvant^®^SOE for its potential to improve the immunogenicity of the FMDV vaccine. The purified inactivated A22 Iraq antigen was emulsified with CAvant^®^SOE to prepare the vaccine formulation. Here we demonstrated that CAvant^®^SOE plays a pivotal role as an immune-enhancing adjuvant that simultaneously induces the inactivated whole antigen vaccines derived strong humoral and cell-mediated immune responses, and effectively induce protective immunity in pigs.

## 2. Materials and Methods

### 2.1. Preparation of the Vaccines

The inactivated and concentrated FMD A22 Iraq virus supplied by the Animal and Plant Quarantine Agency (Korea) was used to manufacture the experimental vaccines. The concentrated A22 Iraq antigens were diluted with a Tris-NaCl buffer (with a pH of 7.6) and then added to CAvant^®^SOE (CAVAC, Daejeon, Korea), Montanide ISA 201 (Seppic, Castres, France). The ratio of the aqueous antigen to the oil adjuvant was 35:65 for CAvant^®^SOE and 50:50 for ISA 201 (volume *v*/*v*). The CAvant^®^SOE adjuvant mixture was homogenized at 6000 rpm for 5 min to form a water-in-oil blend. The ISA 201 adjuvant mixture was stirred at 500 rpm for 10 min at 30 °C to form a water-in-oil-in-water blend. The stability of the vaccines was tested by the drop test method [31,32]. The vaccines were kept refrigerated at 4 °C.

### 2.2. Immunization in Mice

**Figure 1:**
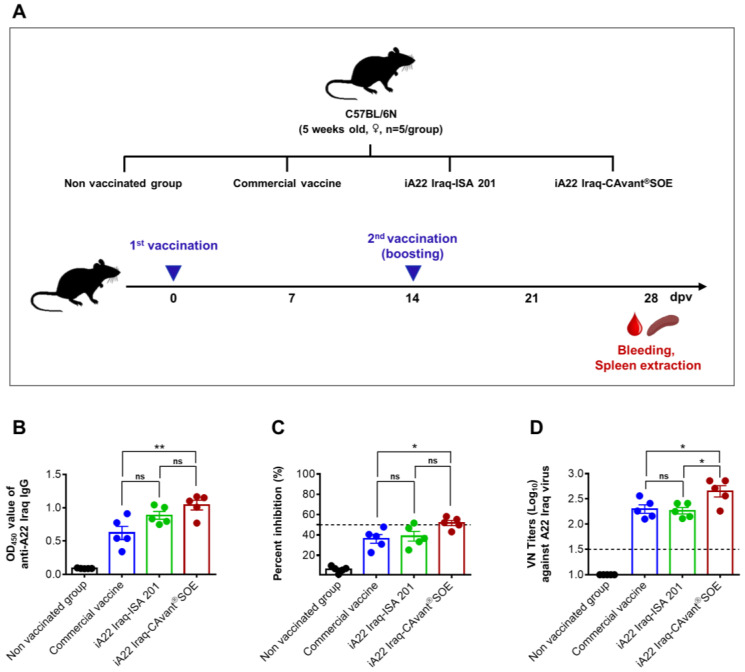
Effect of the CAvant^®^SOE adjuvant on antigen-specific humoral immune response and serum VN titers in mice. (**A**) Schematic depiction of experiment strategy. Mice were intramuscularly immunized twice at 0 and 2 weeks with the commercial vaccine or iA22 Iraq-ISA 201 or iA22Iraq-CAvant^®^SOE and mice without immunization were maintained as a control group (*n* = 5/group). Blood and spleens were taken at 28 days post-vaccination (dpv). (**B**) A22 Iraq-specific total IgG titers by indirect ELISA. (**C**) Type A SP specific antibody titers by type A FMDV SP ELISA. Percent inhibition (PI) > 50 was considered the cutoff of a positive reaction (**D**) serum virus-neutralizing (VN) antibody titers (log10) to the A22 Iraq strain. The values are presented as mean ± SE. Statistical analyses were performed using ANOVA followed by Tukey’s post hoc test. ^ns^ *p* > 0.05, * *p* < 0.05, ** *p* < 0.01.

Groups of 5-week-old female C57BL/6 mice (*n* = 5) were vaccinated via intramuscular injection on 0 and 14 days post-vaccination (dpv) with the 100 µL (1/10 of the 1 mL/dose) of commercial trivalent vaccine (Merial, France) or 1 μg of inactivated A22 Iraq antigen emulsified with ISA 201 (iA22 Iraq-ISA 201) or 1 μg of inactivated A22 Iraq antigen emulsified with CAvant^®^SOE (iA22 Iraq-CAvant^®^SOE). The control group of mice was maintained without vaccination. The serum and spleens were collected at 28 dpv as depicted in Figure 1A.

### 2.3. Immunization and FMDV Challenge in Pigs

To investigate the CAvant^®^SOE mediated host defense against FMDV infection, eight FMDV-specific antibody-negative pigs (10 weeks old) were divided into two test groups (iA22 Iraq-ISA 201 and iA22 Iraq CAvant^®^SOE) and an unvaccinated control group. Test groups were vaccinated intramuscularly with an experimental vaccine containing 10 µg of A22 Iraq antigen per dose. At four weeks’ post-vaccination, the pigs were challenged with 10^5^ TCID_50_/0.1 mL of A/SKR/GP/2018 (GenBank accession no. MK463492.1) via intradermal injection on the heel bulb. Oral swab samples were collected daily from 0 to 8 days post-challenge (dpc) using the BDTM Universal Viral Transport Kit (BD Biosciences). Blood samples were collected on −28, −14, 0, 2, 4, 6, and 8 dpc. The clinical symptoms of each challenged group were continuously observed over eight dpc (Figure 3A).

Clinical score was determined by summing points as follows with a maximum of 17 points: (i) an elevated body temperature of 40 °C (1 point), 40.5 °C (2 points), or 41 °C (3 points); (ii) no food intake, and food leftover from the day before (1 point); (iii) lameness or reluctance to stand (1 point); (iv) Decreased activity and depressed (0.5 point), or convulsion and not standing on the affected foot (1 point); (v) vesicles on the feet, dependent on the number of feet affected with a maximum of 8 points; and (vi) visible mouth lesions on the tongue (1 point), lips (1 point), or snout (1 point) [33,34,35].

### 2.4. Enzyme-Linked Immunosorbent Assay (ELISA)

For the detection of structural protein (SP) antibodies in sera, PrioCHECK FMDV type A ELISA (Prionics AG, Switzerland) was performed according to the manufacturer’s guidelines. The optical density (OD) value at 450 nm was converted to the percent inhibition (PI) value. According to the manufacture’s recommendation cut–off value of >50% PI was considered as a threshold value for seroconversion. For the detection of antigen-specific antibodies, an in-house indirect ELISA was adopted. Briefly, 96 well immune plates (Nunc, Denmark) were coated with 500 ng/well of A22 Iraq-specific peptide overnight at 4 °C. Then wells were washed with wash buffer 3 times and antigen-coated wells were blocked with 10% skim milk for 2 h at room temperature. Then wells were washed again as the previous washing step followed by incubation of 100 μL diluted sera sample (1:200 in 2% skim milk) for 2 h at 37 °C. Then wells were subjected to the previous washing step, followed by incubation with 100 μL of HRP-conjugated goat anti-mouse immunoglobulins (IgG, IgG1, IgG2a, 1:3000, Sigma, St. Louis, MO, USA) for 2 h at 37 °C. Followed by another round of washing, then plates were reacted with 100 μL of TMB substrate solution (BD Bioscience, USA) for 15 min and stop by 50 μL of 2N H_2_SO_4_. Absorbance was measured at 450 nm using Apollo LB 913 ELISA reader (BERTHOLD Technologies, Oak Ridge, TN, USA).

### 2.5. Splenocytes Isolation and Cell-Mediated Immune Responses

For the analysis of antigen-specific T cell-mediated immune responses, ELISPOT plates (BD Bioscience, East Rutherford, Piscataway, NJ, USA) were coated with anti-mouse IFN-γ or IL-4 capture antibodies and incubated at 4 °C. The plates were blocked with complete RPMI 1640 medium containing 10% fetal bovine serum (Gibco, Waltham, MA, USA), in RT for 1 h. Freshly isolated splenocytes were added at 1 × 10^6^ cells/well in media containing the 10 μg/well of A22 Iraq peptide (Table 1), 1 μg/well of phytohemagglutinin (positive control), or only medium (negative control). After 24 h incubation at 37 °C and 5% CO_2_, the plates were added sequentially with biotinylated anti-mouse IFN-γ and IL-4 antibodies, streptavidin-HRP, and substrate solution. Finally, the plates were washed with distilled water and dried for two hours in the dark. Spots were counted using an Immuno Scan Entry Analyzer (Cellular Technology Ltd., Shaker Heights, OH, USA).

### 2.6. Virus Neutralization Test

Titers of neutralizing antibodies in the serum were analyzed via a virus neutralization test with LF-BK cells. Serum samples were collected from the animals after vaccinations and virus challenge. The collected serum samples were heat-inactivated at 56 °C for 30 min and stored at −20 °C until tests were performed.

Two-fold serial dilutions of sera samples were prepared. The diluted serum samples were then incubated with FMDV 100 TCID50/0.1 mL for one hour at 37 °C. After one hour, the LF-BK cell suspension was added to all wells and incubated for three days. The endpoint titers were determined using the results of the cytopathic effect formation, which were calculated as the reciprocal log10 of the highest dilution that neutralized 100 TCID50 of FMDV in 50% of the wells [36].

### 2.7. Analysis of FMD Replication in Pigs

Real-time RT-PCR was performed on serum and swab samples of the challenged animals. Viral RNA was extracted using the QIAamp 96 DNA QIAcube HT Kit (QIAGEN, Germany) according to the manufacturer’s protocol. Real-time RT-PCR was conducted using the AccuPower FMDV Real-time RT-PCR MasterMix Kit (BIONEER, Daejeon, Korea) according to the manufacturer’s instructions. The CFX96 Real-Time PCR Detection System (Bio-Rad, Hercules, CA, USA) was used for virus quantification.

### 2.8. Statistical Analysis

Statistical analysis was performed using GraphPad Prism version 6 (GraphPad Software, San Diego, CA, USA) to examine the immunogenicity and protective effects of the vaccines. All quantitative data were expressed as the mean ± standard error (SEM). Between groups, statistical significance was assessed using two-way ANOVA followed by Tukey’s post hoc test or one-way ANOVA followed by Tukey’s post hoc test; *p*-values of less than 0.05 were considered as a statistical difference between groups.

## 3. Results

### 3.1. CAvant^®^SOE Enhances FMDV-Specific Humoral Immune Responses in Mice

Clinical protection against FMD correlates with humoral immunity, which is used as an indirect tool to measure the potency of FMD vaccines based on serological responses after vaccination [37]. To investigate the effect of CAvant^®^SOE on antigen-specific humoral immune responses, we immunized three groups of mice (intramuscularly) on 0 and 14 dpv with a commercial vaccine or iA22 Iraq-ISA 201 or iA22-CAvant^®^SOE. A control group was not vaccinated (Figure 1A). Blood samples collected at 28 dpv were tested in an in-house indirect ELISA, type A SP ELISA, and virus neutralization test (VNT). Higher levels of antigen-specific total IgG antibody responses were detected in the commercial vaccine, iA22 Iraq-ISA 201, and iA22Iraq-CAvant^®^SOE (Figure 1B). A similar pattern of results was observed in the FMDV type A SP ELISA assay, in which mice immunized with CAvant^®^SOE-adjuvanted A22 Iraq achieved seropositivity concerning mean present inhibition (PI > 50) antibody titers (Figure 1C). The VNT results exhibited better agreement with the ELISA results, iA22 Iraq-CAvant^®^SOE group exhibited high potential for the production of neutralizing antibodies compared to the commercial vaccine group and iA22 Iraq-ISA 201 group (Figure 1D). These results suggest that adjuvant CAvant^®^SOE induces effective humoral immune responses when formulated with inactivated FMDV vaccine antigens.

### 3.2. CAvant^®^SOE Balances FMDV-Specific Th1 and Th2 Immune Responses in Mice

**Figure 2:**
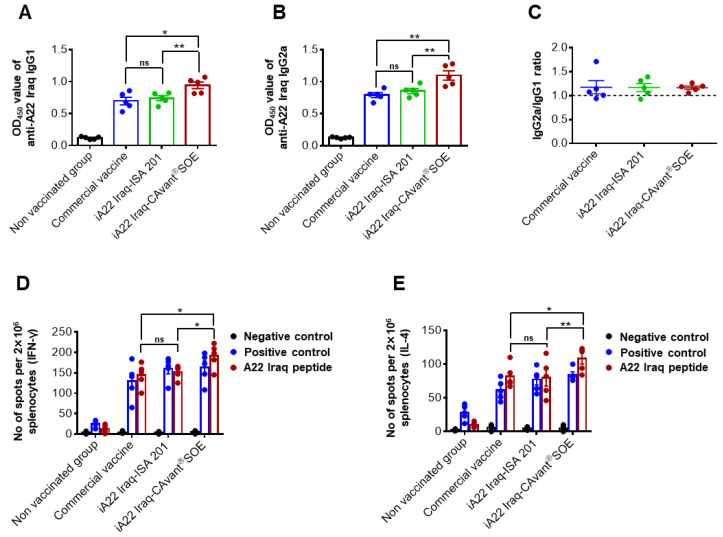
Effect of the CAvant^®^SOE adjuvant on antigen-specific humoral and cell-mediated immune response in mice. Mice were intramuscularly immunized twice at 0 and 2 weeks with the commercial vaccine or A22 Iraq-ISA 201 or A22Iraq-CAvant^®^SOE and mice without immunization were maintained as a control group (*n* = 5/group). Blood and spleens were taken 28 days after vaccination. (**A**) A22 Iraq-specific IgG1, (**B**) IgG2a were measured by indirect ELISA and (**C**) IgG2a/IgG1 ratio was calculated. The number of A22 Iraq-specific (**D**) IFN-γ spot forming units and (**E**) IL-4 spot forming units were determined using enzyme-linked immunosorbent spot (ELISPOT) assay. The values are presented as mean ± SE. Statistical analyses were performed using one-way ANOVA or two-way ANOVA with Tukey’s multiple comparisons test. ^ns^ *p* > 0.05, * *p* < 0.05, ** *p* < 0.01.

The balance between Th1/Th2 immune responses is important for equivalence between antibody and cell-mediated immune responses. The antibody isotype response may reflect the type of immune activation (Th1 or Th2 type) in vivo [38,39]. Thus, we examined antigen-specific IgG isotypes in an indirect ELISA assay. Higher and balance levels of antigen-specific IgG1 and IgG2a antibody responses were detected in the iA22 Iraq-CAvant^®^SOE vaccination group which were higher compared to the commercial vaccine and iA22 Iraq-ISA 201 vaccination groups (Figure 2A–C).

Splenocytes were isolated at 28 dpv and subjected to ELISPOT assays to quantify antigen-specific interferon (IFN)-γ- and IL-4-secreting cells. IFN-γ is a representative Th1 cytokine that is also expressed by cytotoxic T lymphocytes, whereas IL-4 is a Th2 cytokine. Splenocytes were stimulated with the A22 Iraq peptide. Mice immunized with iA22-CAvant^®^SOE show higher numbers of IFN-γ- and IL-4-secreting splenocytes than the commercial vaccine and iA22 Iraq-ISA 201 vaccination groups (Figure 2D,E). These results suggest that adjuvant CAvant^®^SOE improves the balance between humoral and cell-mediated immune responses of the FMDV vaccine antigens.

### 3.3. The CAvant^®^SOE Adjuvant Increases Protection from Challenge with FMDV in Pigs

**Figure 3:**
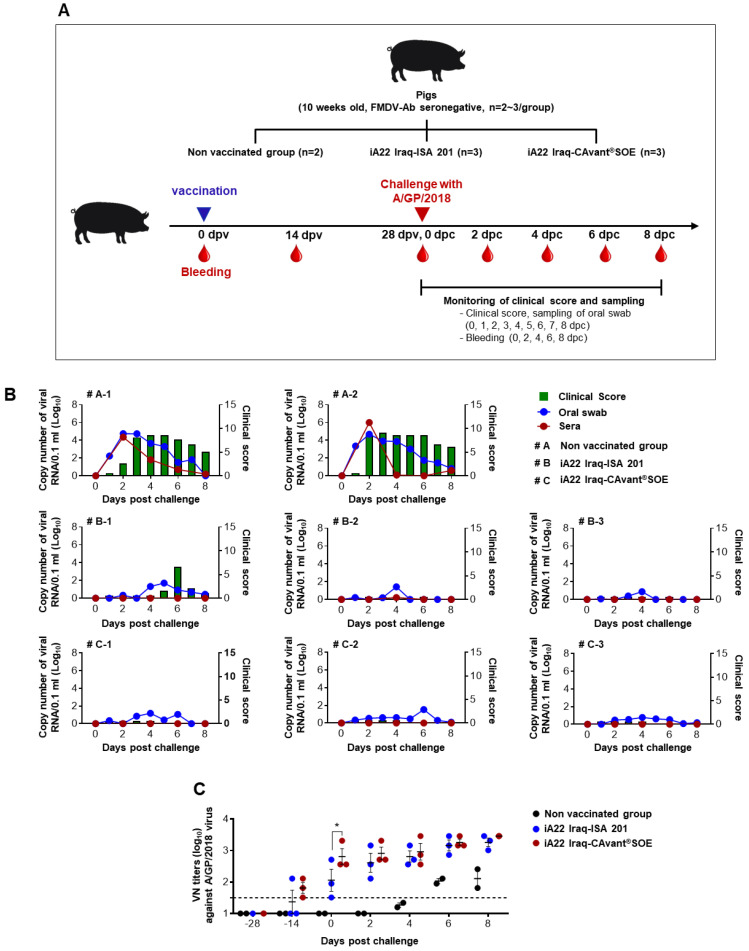
Evaluation of protective efficacy following challenge with the heterologous FMD virus in pigs. (**A**) Schematic depiction of experiment strategy. Pigs were intramuscularly immunized with a single dose of A22 Iraq-ISA 201 or A22 Iraq-CAvant^®^SOE and pigs without immunization maintain as a control group. Blood samples were collected and challenged with A/SKR/GP/2018 strain at 28 dpv. (**B**) The changes in clinical score and FMDV RNA levels in sera and mouth swab by qPCR from 0 to 8 days after challenge. (**C**) VN titer to the A/SKR/GP/2018 strain. VN antibody titer value of >1:32 (1.5 log10) was considered the cutoff of protectable value. The values are presented as mean ± SE. Statistical analyses were performed using two-way ANOVA with Tukey’s multiple comparisons test. * *p* < 0.05.

Finally, to investigate the protection offered by the iA22 Iraq-CAvant^®^SOE vaccine, three groups of pigs were vaccinated intramuscularly with inactivated A22 Iraq virus emulsified with CAvant^®^SOE or ISA 201, the control group was not vaccinated. Pigs were challenged with A/SKR/GP/2018 Asia topotype at 28 dpv. Following the challenge, several FMD parameters were analyzed, including clinical signs, viremia in sera, and oral swab. The non-vaccinated group starts to show severe clinical signs at 2 dpc which persisted until 8 dpc and a high level of the virus was detected in sera and oral swabs. In the ISA 201-adjuvanted vaccine group, one out of three animals show moderate clinical signs at late time (5 dpc) and persist only for 3 days and recovered at 8 dpc. Contrastingly, all animals in the CAvant^®^SOE-adjuvanted vaccine immunized group showed clinically protective results with a low mean clinical score (<0.4). The viremia was not detected in the sera and virus shedding was barely detected, in both iA22 Iraq-ISA 201 and iA22-CAvant^®^SOE vaccination groups (Figure 3B).

The serum neutralizing antibody kinetics for the A/SKR/GP/2018 were investigated from pre-immune status to 8 days post virus challenge. All pigs in iA22-CAvant^®^SOE vaccination groups were seropositive at 14 dpv. While one out of three pigs in iA22 Iraq-ISA 201 vaccination group were seropositive at 14 dpv. Though all pigs in both iA22 Iraq-ISA 201 and iA22-CAvant^®^SOE vaccination groups were seropositive at 28 dpv pigs in iA22- CAvant^®^SOE vaccination groups show significantly higher VN titers than pigs in iA22 Iraq-ISA 201 vaccination group (Figure 3C). Overall, the results suggest that the CAvant^®^SOE-adjuvanted vaccine effectively protects pigs against FMD.

## 4. Discussion

Nationwide mass vaccination in enzootic areas seeks to prevent, control, and, eventually, eradicate FMD. FMD-free countries rely on high potency “emergency” vaccines to control outbreak situations. To achieve all of these objectives, vaccine efficacy is crucial and may best be achieved by as few vaccinations as possible, with rapid induction of an immune response to achieve protection [14]. The adjuvant is an integral component of FMD vaccines; the adjuvant increases vaccine efficacy and provides an important means for achieving both early and long-lasting immunity and protection. Moreover, adjuvants might reduce the amount of antigen required or the round of immunizations necessary to induce a protective immune response. In general, adjuvants are chemical substances that boost the immune response against a particular antigen [18]. Hence, much research is devoted to discovering and developing novel potent adjuvants that will increase the efficacy of FMD vaccines.

Mineral oil-based adjuvants have been used widely as adjuvants for FMD vaccines and other veterinary vaccines for over 20 years [29]. Numerous reports show that oil-based vaccine formulations show greater efficacy (i.e., stronger and longer-lasting immune responses as well as rapid onset of protection) than traditional alum/saponin-based vaccines [21,23,27,28]. In particular, the most widely used oil adjuvant the Montanide ISA 206 certainly more effective than traditional alum/saponin-based adjuvant in terms of generating higher and long-lasting immune responses [18]. However, less efficacy of ISA 206 in inducing cell-mediated immune response and consequent insufficient protective efficacy in cattle against FMD virus had been frequently reported in recent years [14,18,26]. The Montanide ISA 201 is the most recent water-in-oil-in-water candidate belonging to the ISA adjuvant series. It is an advanced version of ISA 206 to improve the cell-mediated immune responses, and better protective efficacy in cattle and pigs than ISA 206 [14,18,29,30].

CAvant^®^SOE is a novel W/O emulsion type adjuvant containing a proprietary emulsified component. CAvant^®^SOE, which has the advantage of W/O emulsion, induces stronger protective immunity compared to aqueous vaccines. The W/O emulsion platform can have a variety of effects on vaccine biological activity by modulating antigen delivery to APCs or having an intrinsic adjuvant effect through direct stimulation of immune cells [40,41]. In addition, CAvant^®^SOE can have immunomodulatory properties beyond their ability to trigger global immune stimulation, by directing T helper (Th) 1 and Th2 response. The protective immunity to antigen is attributed to the induction of neutralizing antibodies.

The induction of specific humoral immunity is the main priority of any vaccine. Protective immunity against FMDV is usually attributed to the induction of neutralizing antibodies [42,43]. Thus, for an adjuvant to be effective, it should generate neutralizing antibody responses more rapidly, at higher titers, and for a longer duration [14]. In particular, CAvant^®^SOE adjuvant is effective in inducing antigen-specific total IgG antibody titers and neutralizing antibody responses against FMDV.

The subclass of immunoglobulin that is induced after immunization is an indirect measure of the relative contribution of Th2-type versus Th1-type cytokines [44]. The production of IgG1 isotype is generally reflected Th2 cytokines which promote humoral immune responses, whereas IgG2a isotypes indicate the involvement of Th1-type cytokines which promote the generation of cellular immune responses. The ratio of these two isotypes indicates the type of immune response generated against a given antigen [44,45]. We, therefore, examined the ratios of IgG2a to IgG1 antibody generated in response to vaccination, iA22-CAvant^®^SOE vaccination groups show mixed Th1/Th2 immune responses, indicating that CAvant^®^SOE adjuvant-induced higher and balanced humoral and cellular immune responses. This data show clear agreement with Th2 (IL-4) and Th1 (IFN-γ) cytokine levels in ELISPOT assay, further confirmed that CAvant^®^SOE effectively induces both Th1 and Th2 responses.

Better clinical protection against live virus challenge is the main objective of any vaccine study. The results of our work show that CAvant^®^SOE promotes effective protection against heterologous virus challenges. According to the FMD vaccine evaluation criteria in Korea, host defense is possible when the VN titer induced by commercial vaccination is >1.5 (log10). In this study, a strong relationship was observed between VN antibody titers and clinical protection. All pigs vaccinated with CAvant^®^SOE adjuvanted vaccine show VN titers as high as >2.4 (log10) at 28 dpv and completely protect from the FMDV challenge.

## 5. Conclusions

Taken together, the findings reported herein indicate that the CAvant^®^SOE adjuvant-based FMDV vaccine provides better humoral and cell-mediated immune responses and effective protection against FMDV in pigs. Further research should be conducted to prove the effect of CAvant^®^SOE adjuvant on the effective early and long-term immunity with more animal numbers in the FMD vaccines.

## Figures and Tables

**Table 1:** Peptide used for ELISA and ELISPOT.

Protein	aa Position	aa Sequence
A22 Iraq VP1	139–158	GGTGRRGDLGPLAARVAAQL

aa: Amino acid.

## Data Availability

All datasets generated for this study are included in the article.
